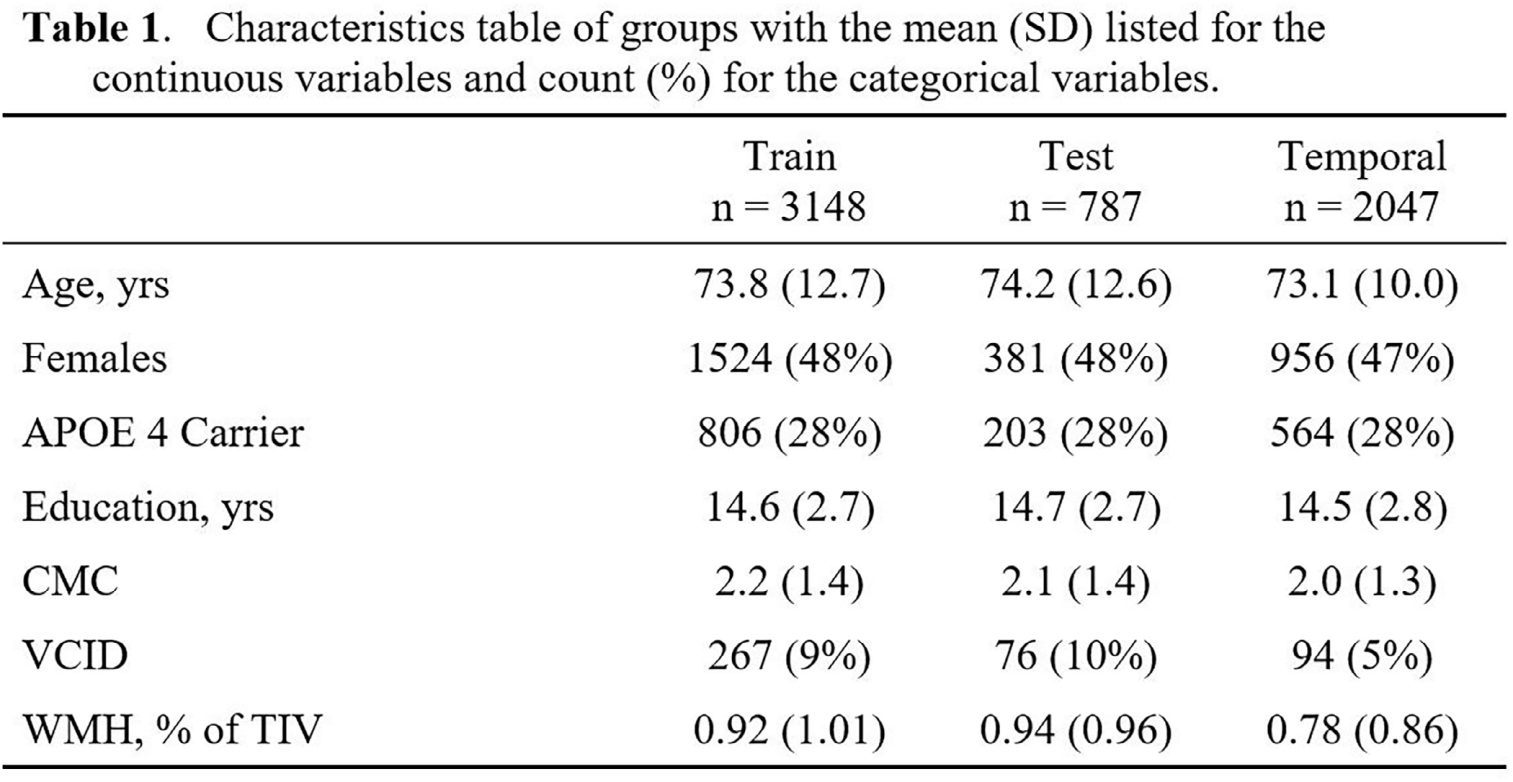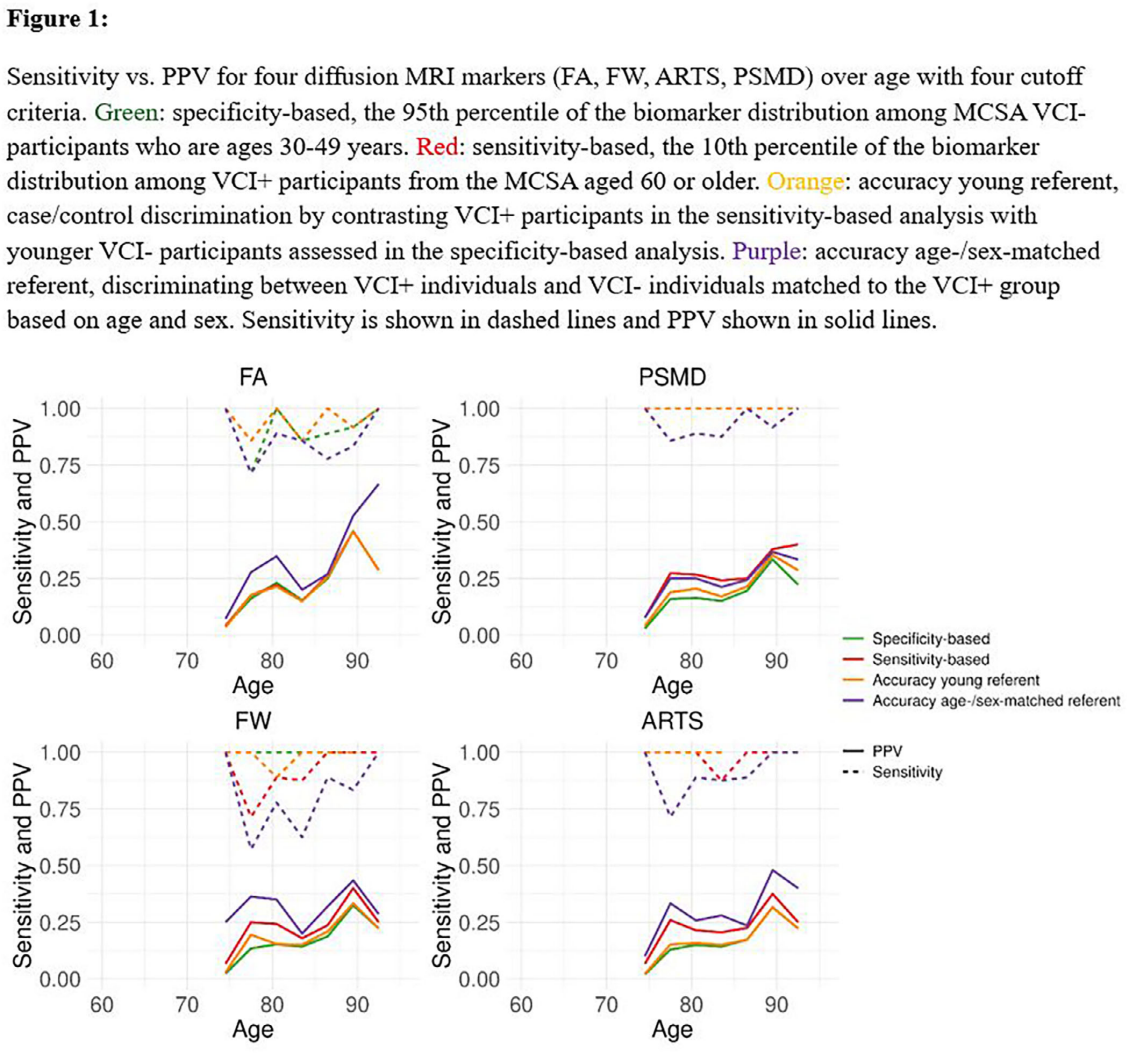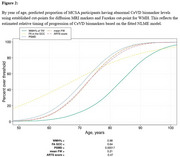# Early & late cerebrovascular biomarkers: Operationalization of cut‐points and temporal progression

**DOI:** 10.1002/alz70856_104352

**Published:** 2025-12-26

**Authors:** Mingzhao Hu

**Affiliations:** ^1^ Department of Quantitative Health Sciences, Mayo Clinic, Rochester, MN, USA

## Abstract

**Background:**

Cerebrovascular disease (CeVD), a common co‐pathology with Alzheimer's Disease, has not been sufficiently operationalized, limiting its usage for accurate estimation of vascular contributions to cognitive impairment (VCI). The proposed CeVD biomarker classification uses more recently developed diffusion MRI markers (fractional anisotropy of Genu (FA), free water (FW), peak width of skeletonized mean diffusivity (PSMD), and ARTS score (ARTS)) as surrogates of early CeVD changes and white matter hyperintensities (WMH) as surrogate of late CeVD changes. Our primary goal was to characterize the temporal progression of these biomarkers to enhance CeVD biomarker staging. Our secondary goal was to establish cut‐points for normal/abnormal CeVD biomarkers.

**Methods:**

Using CeVD biomarker data from 3,953 Mayo Clinic Study of Aging participants (split 80‐20 for training‐testing with training used for cut‐point estimation), we classified participants into VCI+/VCI‐ (Fazekas≥2 and Clinical Dementia Rating (CDR)≥0.5/not). We evaluated cut‐points using four criteria: specificity‐based, sensitivity‐based, accuracy young referent (VCI+ vs. 30‐49 yrs VCI‐), and accuracy age‐/sex‐matched referent (VCI+ vs. age‐/sex‐matched VCI‐). After selecting a cut‐point based on sensitivity and positive predictive value (PPV), we used nonlinear mixed‐effects (NLME) models to examine temporal progression in 2,047 participants (aged 43.39‐92.51 years, 47% female) with longitudinal data incorporating participant‐specific time shifts, correlations between random effects of each biomarker, and covariates (age, sex, vascular risk, education, *APOE4* status).

**Results:**

The accuracy young referent criterion produced the best sensitivity‐PPV tradeoff across all diffusion MRI markers (Figure 1). Diffusion MRI biomarkers consistently progressed 10 years earlier than WMH (Figure 2). Whole‐brain diffusion MRI measures (FW, PSMD, ARTS) were highly correlated with WMH, showing synchronized progression curves with a time offset. The regional genu measure (interhemispheric disconnection) progressed earlier with a steadier slope across decades and had comparatively lower correlation with WMH.

**Conclusions:**

We developed cut‐points for diffusion MRI markers using classification of VCI based on Fazekas scale and CDR. Cut‐points from the accuracy young referent (VCI+ vs. 30‐49 yrs VCI‐) outperformed the accuracy age‐/sex‐matched referent (VCI+ vs. age‐/sex‐matched VCI‐), suggesting that older VCI‐ individuals may have unmeasured CeVD changes. Diffusion MRI markers progressed earlier relative to WMH, highlighting their potential for early detection, improved clinical assessment, and targeted interventions.